# No Intra-Locus Sexual Conflict over Reproductive Fitness or Ageing in Field Crickets

**DOI:** 10.1371/journal.pone.0000155

**Published:** 2007-01-17

**Authors:** Felix Zajitschek, John Hunt, Susanne R.K. Zajitschek, Michael D. Jennions, Robert Brooks

**Affiliations:** 1 School of Biological, Earth and Environmental Sciences, The University of New South Wales, Sydney, Australia; 2 Centre for Ecology and Conservation, University of Exeter in Cornwall, Penryn, United Kingdom; 3 School of Botany and Zoology, Australian National University, Canberra, Australia; University of Oxford, United Kingdom

## Abstract

Differences in the ways in which males and females maximize evolutionary fitness can lead to intra-locus sexual conflict in which genes delivering fitness benefits to one sex are costly when expressed in the other. Trade-offs between current reproductive effort and future reproduction and survival are fundamental to the evolutionary biology of ageing. This leads to the prediction that sex differences in the optimization of age-dependent reproductive effort may generate intra-locus sexual conflict over ageing rates. Here we test for intra-locus sexual conflict over age-dependent reproductive effort and longevity in the black field cricket, *Teleogryllus commodus*. Using a half-sib breeding design, we show that the most important components of male and female reproductive effort (male calling effort and the number of eggs laid by females) were positively genetically correlated, especially in early adulthood. However, the genetic relationships between longevity and reproductive effort were different for males and females, leading to low genetic covariation between male and female longevity. The apparent absence of intra-locus sexual conflict over ageing suggests that male and female longevity can evolve largely independently of one another.

## Introduction

There is a growing appreciation that the conflict of evolutionary interests between males and females is a powerful and near-ubiquitous evolutionary force [1]–[3]. There are two broad ways in which sexual conflict can impact on evolution. First, when a particular interaction between a male and a female has different optimal outcomes for the two players, they are said to be in inter-locus conflict [2]. Such inter-locus conflict provides the basis for antagonistic coevolution between males and females [2], [4]. Second, when selection favours different optimal trait values in males and females (i.e. selection is sex-specific), there is conflicting selection on the same body of genetic variation, depending on whether the genes are expressed in a male or a female. In polygenic traits, this intra-locus sexual conflict [2] is mediated by the strength of the genetic correlation between the traits expressed in male and female (i.e., the inter-sexual genetic correlation), and constrains the evolution of sexual dimorphism [5], [6].

Differences in longevity between males and females are widespread [7], [8]. In mammals, the largest such differences are in species with the greatest sexual size dimorphism, suggesting that more intense sexual selection increases relative male mortality [7], [9]. This interpretation is consistent with sexual selection theory in which both direct male-male competition and sexual advertising are extremely costly [10], [11], leading to reduced male lifespan. It was recently argued that sexual conflict might be an important source of sex differences in ageing [8], [12]. The prediction that males directly influence the longevity of their mates has been verified [13], confirming a role for inter-locus sexual conflict in the evolution of ageing. However, the prediction that sex differences in the optimal timing and relative costliness of reproductive effort should lead to sex-specific selection on the relationship between lifespan and evolutionary fitness, and thus to intra-locus conflict over optimal male and female ageing rates remains to be tested.

Here we test a number of predictions regarding the relationship between sex-specific selection on age-dependent reproductive effort and the evolution of male and female ageing. These predictions arise from the antagonistic pleiotropy theory of ageing in which genes that have beneficial effects on components of fitness early in life have antagonistic deleterious effects when expressed at old ages [14]–[16]. Ageing is manifested not only in patterns of longevity/mortality, but also in age-dependent declines in reproductive effort [17]–[19]. Moreover, a primary determinant of both of these forms of ageing is the age-dependent pattern of reproductive effort in early adulthood [20]–[22]. Studies exploring the potential for intra-locus sexual conflict over fitness or ageing should therefore test the following predictions. (1) Current intra-locus sexual conflict should result in a negative genetic correlation between male and female fitness, or at least lifetime reproductive success. Such a correlation could be mediated by (2) sex-specific differences in the trade-off between reproductive effort and lifespan, and (3) between early and late reproductive effort. (4) Strong negative genetic correlations between male and female longevity would further indicate intra-locus sexual conflict over age-dependent reproductive effort.

Predictions 1–4 above relate to the signatures of current intra-locus sexual conflict. The alternative, in which contemporary intra-locus sexual conflict is weak, would be characterised by strong positive genetic correlations and similarities in the age-dependent ways in which males and females invest in reproduction. A third possibility arises from the fact that intra-locus sexual conflict should select for non-Mendelian genetic mechanisms that reduce the inter-sexual genetic correlation [5], [6], [23]–[26]. A signature of long-term historic intra-locus sexual conflict under this scenario would be inter-sexual genetic correlations that tend toward zero.

So far, few studies have found direct support for negative genetic correlations between male and female reproductive fitness [27]–[29]. We are not aware of any studies that have found evidence for a lack of intra-locus sexual conflict over fitness. Despite ample support for the antagonistic pleiotropy theory of ageing within sexes [15], [20], [30]–[32], there have been no explicit attempts to resolve the effects of sex-specific differences in reproductive trade-offs on male and female ageing.

Here we explore the quantitative genetic relationships between reproductive effort and ageing between and within males and females of the native Australian black field cricket, *Teleogryllus commodus*. Females are polyandrous, exhibit strong mate choice based on male call traits, and there is good evidence for sexual conflict over insemination [33]. We can accurately quantify male and female reproductive effort [34] which allows us to test for intra-locus sexual conflict over the timing of investment into reproductive effort and longevity.

The major determinant of male mating success in *T. commodus* is the long-distance advertisement call [34], [35]. Males fed a high quality diet die sooner than males on poorer diets and this shorter lifespan is phenotypically associated with the onset of calling at a younger age and more time spent calling per night [34]. By contrast, female *T. commodus* live longer on a high quality diet than on a low quality diet [34]. This phenotypic trade-off suggests that there are differences in how males and females allocate resources to current versus future reproduction. The associated effects on longevity raise the intriguing possibility of intra-locus sexual conflict.

We used a full-sib/half-sib breeding design to estimate the genetic correlations necessary to test our four predictions of how sex differences in age-dependent reproductive effort may lead to intra-locus sexual conflict over ageing. First, we test for intra-locus sexual conflict over reproductive fitness by estimating the genetic correlation between lifetime male calling effort and lifetime female fecundity. We then estimate intra-sexual genetic correlations between reproductive effort and lifespan, and between early and late reproductive to test the hypothesis that relationships between these fitness components differ between the sexes, thus generating intra-locus sexual conflict over reproduction or longevity. Last, we estimate the genetic correlation between male and female longevity, as a strong negative inter-sexual genetic correlation for longevity would be an indication for differences in the age-dependent pattern of how males and females invest into reproduction.

## Results

**Figure 1:**
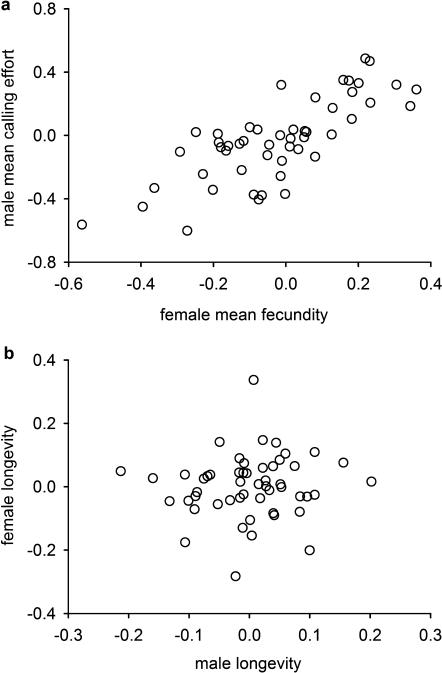
Inter-sexual genetic correlations between (a) male mean calling effort and female mean fecundity and (b) male and female longevity.

Male mean calling effort and female mean fecundity showed a strong positive genetic correlation (Fig 1a and Table 1, *r_A_* = 0.64±0.26 s.e.). When we assess total calling effort and fecundity the positive genetic correlation is even stronger (Table 1, *r_A_* = 0.93±0.24 s.e.). However, sex differences in the trade-off between reproductive effort and longevity resulted in a comparatively low genetic correlation between male and female longevity (Fig 1b and Table 1, *r_A_* = 0.29±0.39 s.e.).

**Table 1:**
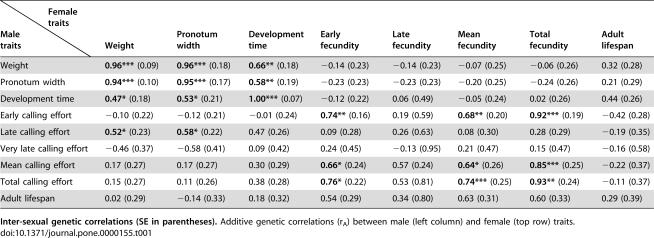
Inter-sexual Genetic Correlations for Life-History Traits.

Female traits Male traits	Weight	Pronotum width	Development time	Early fecundity	Late fecundity	Mean fecundity	Total fecundity	Adult lifespan
Weight	0.96*** (0.09)	0.96*** (0.18)	0.66** (0.18)	−0.14 (0.23)	−0.14 (0.23)	−0.07 (0.25)	−0.06 (0.26)	0.32 (0.28)
Pronotum width	0.94*** (0.10)	0.95*** (0.17)	0.58** (0.19)	−0.23 (0.23)	−0.23 (0.23)	−0.20 (0.25)	−0.24 (0.26)	0.21 (0.29)
Development time	0.47* (0.18)	0.53* (0.21)	1.00*** (0.07)	−0.12 (0.22)	0.06 (0.49)	−0.05 (0.24)	0.02 (0.26)	0.44 (0.26)
Early calling effort	−0.10 (0.22)	−0.12 (0.21)	−0.01 (0.24)	0.74** (0.16)	0.19 (0.59)	0.68** (0.20)	0.92*** (0.19)	−0.42 (0.28)
Late calling effort	0.52* (0.23)	0.58* (0.22)	0.47 (0.26)	0.09 (0.28)	0.26 (0.63)	0.08 (0.30)	0.28 (0.29)	−0.19 (0.35)
Very late calling effort	−0.46 (0.37)	−0.58 (0.41)	0.09 (0.42)	0.24 (0.45)	−0.13 (0.95)	0.21 (0.47)	0.15 (0.47)	−0.16 (0.58)
Mean calling effort	0.17 (0.27)	0.17 (0.27)	0.30 (0.29)	0.66* (0.24)	0.57 (0.24)	0.64* (0.26)	0.85*** (0.25)	−0.22 (0.37)
Total calling effort	0.15 (0.27)	0.11 (0.26)	0.38 (0.28)	0.76* (0.22)	0.53 (0.81)	0.74*** (0.25)	0.93** (0.24)	−0.11 (0.37)
Adult lifespan	0.02 (0.29)	−0.14 (0.33)	0.18 (0.32)	0.54 (0.29)	0.34 (0.80)	0.63 (0.31)	0.60 (0.33)	0.29 (0.39)

**Inter-sexual genetic correlations (SE in parentheses).** Additive genetic correlations (r_A_) between male (left column) and female (top row) traits.

**Figure 2:**
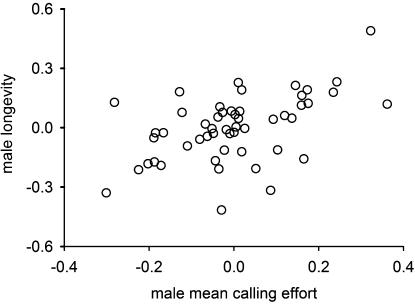
Genetic correlation between male longevity and mean calling effort. Genetic correlations are illustrated as breeding values of the 52 sires for the two traits from a bivariate model.

All measured traits in males, except very late calling effort, were heritable (significantly>0, Table 2). Males showed a positive genetic correlation between early and late reproductive effort (Table 2, *r_A_* = 0.62±0.22 s.e.), and both mean (Fig 2) and total calling effort were positively genetically correlated with male longevity (Table 2, mean calling effort – male longevity: *r_A_* = 0.42±0.33 s.e., total calling effort – male longevity: *r_A_* = 0.52±0.29 s.e.).

**Table 2:**
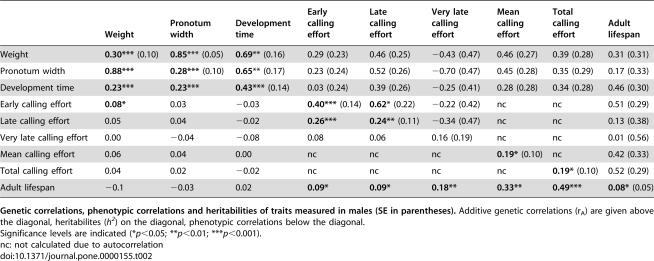
Heritabilities and Genetic and Phenotypic Correlations of Life-History Traits for Males.

	Weight	Pronotum width	Development time	Early calling effort	Late calling effort	Very late calling effort	Mean calling effort	Total calling effort	Adult lifespan
Weight	0.30*** (0.10)	0.85*** (0.05)	0.69** (0.16)	0.29 (0.23)	0.46 (0.25)	−0.43 (0.47)	0.46 (0.27)	0.39 (0.28)	0.31 (0.31)
Pronotum width	0.88***	0.28*** (0.10)	0.65** (0.17)	0.23 (0.24)	0.52 (0.26)	−0.70 (0.47)	0.45 (0.28)	0.35 (0.29)	0.17 (0.33)
Development time	0.23***	0.23***	0.43*** (0.14)	0.03 (0.24)	0.39 (0.26)	−0.25 (0.41)	0.28 (0.28)	0.34 (0.28)	0.46 (0.30)
Early calling effort	0.08*	0.03	−0.03	0.40*** (0.14)	0.62* (0.22)	−0.22 (0.42)	nc	nc	0.51 (0.29)
Late calling effort	0.05	0.04	−0.02	0.26***	0.24** (0.11)	−0.34 (0.47)	nc	nc	0.13 (0.38)
Very late calling effort	0.00	−0.04	−0.08	0.08	0.06	0.16 (0.19)	nc	nc	0.01 (0.56)
Mean calling effort	0.06	0.04	0.00	nc	nc	nc	0.19* (0.10)	nc	0.42 (0.33)
Total calling effort	0.04	0.02	−0.02	nc	nc	nc	nc	0.19* (0.10)	0.52 (0.29)
Adult lifespan	−0.1	−0.03	0.02	0.09*	0.09*	0.18**	0.33**	0.49***	0.08* (0.05)

**Genetic correlations, phenotypic correlations and heritabilities of traits measured in males (SE in parentheses).** Additive genetic correlations (r_A_) are given above the diagonal, heritabilites (*h^2^*) on the diagonal, phenotypic correlations below the diagonal.

Significance levels are indicated (*p<0.05; **p<0.01; ***p<0.001).

nc: not calculated due to autocorrelation

**Figure 3:**
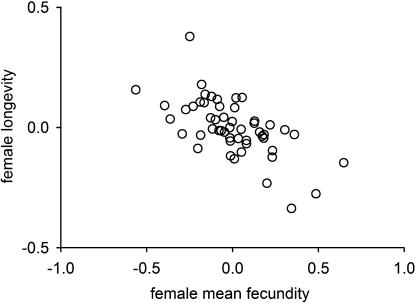
Genetic correlation between female longevity and mean fecundity. Genetic correlations are illustrated as breeding values of the 52 sires for the two traits from a bivariate model.

We found significant heritable variance of all traits measured in females (Table 3). Females also showed a positive genetic correlations between early and late reproductive effort (Table 3, *r_A_* = 0.63±0.62 s.e.). In contrast with males, however, there was strong genetic evidence for antagonistic pleiotropy between fecundity and longevity (Fig 3 and Table 3; mean fecundity *r_A_* = −0.63±0.27 s.e.).

**Table 3:**
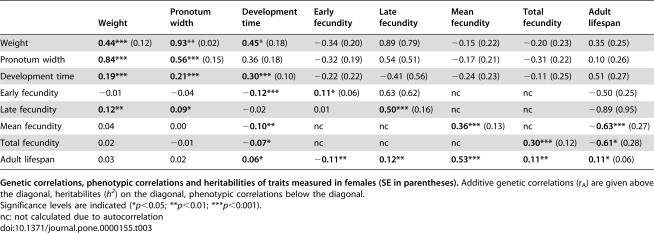
Heritabilities and Genetic and Phenotypic Correlations for the Life-History Traits of Females.

	Weight	Pronotum width	Development time	Early fecundity	Late fecundity	Mean fecundity	Total fecundity	Adult lifespan
Weight	0.44*** (0.12)	0.93** (0.02)	0.45* (0.18)	−0.34 (0.20)	0.89 (0.79)	−0.15 (0.22)	−0.20 (0.23)	0.35 (0.25)
Pronotum width	0.84***	0.56*** (0.15)	0.36 (0.18)	−0.32 (0.19)	0.54 (0.51)	−0.17 (0.21)	−0.31 (0.22)	0.10 (0.26)
Development time	0.19***	0.21***	0.30*** (0.10)	−0.22 (0.22)	−0.41 (0.56)	−0.24 (0.23)	−0.11 (0.25)	0.51 (0.27)
Early fecundity	−0.01	−0.04	−0.12***	0.11* (0.06)	0.63 (0.62)	nc	nc	−0.50 (0.25)
Late fecundity	0.12**	0.09*	−0.02	0.01	0.50*** (0.16)	nc	nc	−0.89 (0.95)
Mean fecundity	0.04	0.00	−0.10**	nc	nc	0.36*** (0.13)	nc	−0.63*** (0.27)
Total fecundity	0.02	−0.01	−0.07*	nc	nc	nc	0.30*** (0.12)	−0.61* (0.28)
Adult lifespan	0.03	0.02	0.06*	−0.11**	0.12**	0.53***	0.11**	0.11* (0.06)

**Genetic correlations, phenotypic correlations and heritabilities of traits measured in females (SE in parentheses).** Additive genetic correlations (r_A_) are given above the diagonal, heritabilites (*h^2^*) on the diagonal, phenotypic correlations below the diagonal.

Significance levels are indicated (*p<0.05; **p<0.01; ***p<0.001).

nc: not calculated due to autocorrelation

The role of reproductive effort in female ageing is further substantiated by the fact that unmated females lived significantly longer than mated females (mean longevity = 55.5 days±0.6 s.e., n = 891 vs. 48.9 days±0. 5 s.e., n = 830; t_1719_ = 8.002, P<0.0001).

## Discussion

We found no evidence of intra-locus sexual conflict over lifetime reproductive effort in *T. commodus*. In fact, there was a strong positive genetic correlation suggesting a common genetic basis for variation in lifetime reproductive effort in males and in females. There was a difference in the way males and females traded off lifespan against reproductive effort, but no evidence for antagonistic pleiotropy between early and late reproductive effort in either sex. Sex-specific longevity was not tightly genetically correlated, suggesting that the evolution of lifespan is unlikely to be constrained by strong negative or positive genetic correlations between the sexes.

Although the form of reproductive effort differs considerably between male and female *T. commodus*, the strong positive genetic correlations between male and female reproductive effort indicate there is little support for intra-locus sexual conflict. On the contrary, sires that produce attractive sons (i.e. males that call a lot) also produce fecund daughters. Our findings demonstrate that intra-locus sexual conflict, while important in some species [27], [28], is not universal. The extent to which intra-locus sexual conflict constrains the evolution of male and female ageing in other species, including those that show intra-locus sexual conflict over fitness [27], [28], remains to be explored.

Females, but not males showed antagonistic pleiotropy between reproduction and longevity. However, in *T. commodus*, the sign of the phenotypic relationship between reproductive effort and longevity is known to be diet-mediated [34], and a recent selection experiment [36] suggests that antagonistic pleiotropy between male calling effort and longevity may occur under some environmental conditions. Given the importance of diet in ageing [37]–[39], and the fact that genetic correlations between life-history traits often vary among environments [40], the complex interactions between diet, age and sex-dependent reproductive trade-offs are an important priority for future study.

Current reproductive effort is predicted by life-history theory, and particularly evolutionary theories of ageing, to trade off against both future reproduction and longevity [41], [42]. Such trade-offs may be manifested as negative genetic correlations between early and late reproductive effort and between reproductive effort (especially early in adulthood) and longevity. We found no evidence for antagonistic pleiotropy between early and late reproductive effort in either sex. This is consistent with the positive pleiotropy between male and female reproduction: good genes for reproductive effort are expressed in both sexes and at all ages.

The weak positive genetic correlation between male and female lifespan is consistent with the results of a previous selection experiment [36]. In that study, four generations of divergent selection on longer male lifespan resulted in approximately zero correlated response in female longevity, whereas selection for shorter male lifespan yielded a strong correlated response in female longevity [36]. Our results appear to fall somewhere in between the effects seen in the divergent selection treatments. The major caveat, however, is that selecting directly on lifespan in males could have led to unwanted selection on other traits, including longevity, in females [20]. This could have happened, for example, through elevated mating rates of females in response to altered male advertising, as male age-specific calling effort and their overall calling-effort also showed correlated responses to selection. Greater longevity was associated with later onset of calling, fewer calls per night and, surprisingly, lower total lifetime calling effort. Female total lifetime fecundity did not show a significant correlated response to selection on male longevity. This implies that the increase in longevity did not affect the effective reproductive lifespan of females. Although selection experiments such as the one by Hunt et al (2006) provide excellent evidence of how traits actually respond to selection, unless we also select on female longevity, this approach does not allow us to formally estimate heritability of female longevity and the genetic correlation between male and female longevity [43].The relatively low inter-sexual genetic correlation in longevity that we find formally indicates that lifespan is to some extent at least free to evolve independently in the two sexes.

In considering our results, there are some possible difficulties with estimating and interpreting genetic correlations in a laboratory setting that have to be considered [44], [45]. Novel environmental effects might bias estimates of genetic covariance toward more positive values. This effect has been shown in empirical studies, but the observed effects were inconsistent in their magnitude [46], [47], and it is unclear how these effects are related to the actual genetic architecture of wild populations [48]. Despite these problems, two arguments mitigate against a novel-environment effect inflating our genetic covariance estimates: firstly, the genetic correlation between fecundity and longevity in females is strong and negative. Novel-environment effects are predicted to manifest as inflated positive genetic correlations between pairs of traits, which is not the case here. Secondly, our estimate of the genetic correlation between male and female longevity is within the range of correlated responses of female longevity to artificial selection on male longevity [36], which are not expected to be strongly influenced by novel environments [49].

In conclusion, our results suggest that intra-locus sexual conflict does not play an important role in the evolution of reproductive effort or ageing in this cricket population. There was a surprisingly strong positive genetic correlation between reproductive effort in the sexes, suggesting considerable pleiotropy between male and female reproduction. Male and female longevity are only weakly genetically correlated, thereby providing considerable scope for independent evolution of lifespan in the sexes. One possibility that raises a prediction for further study, however, is that this weak genetic correlation is a consequence of selection to ameliorate historic intra-locus sexual conflict over ageing. Such selection may have favoured the evolution of genetic mechanisms that support sex-specific inheritance or expression of genes that previously had conflicting effects on male and female longevity, including sex-linkage [23], [25] or epigenetic mechanisms [6], [26]. Although the genetic basis for reproductive effort in males and in females was very similar, the strong genetic correlations between reproductive effort and longevity within each sex were of opposite sign, suggesting that the links between reproductive effort and longevity are likely to be complex.

## Materials and Methods

### Experimental design

The laboratory stock we used originated from near Smith's Lake, New South Wales, Australia. After capture, the stock was bred for four generations in captivity before the start of this experiment. We mated 52 stock males to 7 dams each, resulting in 301 families, as some dams did not produce any offspring. For each family we collected 12 offspring and reared them individually. In total, 86% of animals reached adulthood (n = 3121). We measured nightly calling effort for 3 males per family on day 10, day 25, and between day 60 and 65 after eclosion. Data on field longevity of the same founder population suggests that these ages represent young, old, and very old individuals, as the median longevity found in a capture-mark-recapture study was 10 days after eclosion in males (n = 159), 14 days in females (n = 494), and the longest period between capture and recapture was 58 days in males, and 64 days in females (F. Zajitschek et al., unpublished). In order to concentrate on one of the most important component of attractiveness of male crickets, calling effort [35], [50], we did not allow males in this experiment to mate. Three females per full-sib family were drawn at random and each mated to a different non-experimental stock male at day 10 and to another male at day 25 post-eclosion. They were then allowed to lay eggs in the sand-filled Petri dishes provided for 1 week after the successful transfer of the spermatophore. Eggs were counted to estimate age-dependent fecundity. The remaining females in the family were kept until they died, but never mated. Although mean longevity differed between mated and unmated females, it was strongly positively genetically correlated between mated and unmated females (*r_A_* = 1.34±0.58 s.e.), and thus female longevity is treated as a single trait, with number of matings (1, 2 or 0) fitted as a fixed effect. All experimental and stock crickets were kept on the same diet (Friskies Go-Cat® Senior).

### Statistical analysis

Normally distributed traits were standardized before analysis. Mean number of eggs and mean calling effort were calculated for individuals as the sum of the values of the respective trait, divided by the number of measures at which the animal was alive. There was no suitable transformation available to normalize measures of calling effort and egg counts, because in many cases males did not call and females did not lay eggs, resulting in an excess of zeros in the dataset. Because of the extremely skewed distribution we ranked the trait values, using a random number generator to break ties. We then numbered the values in rank order from 1/n to 0.999 and used the NORMSINV function in Microsoft Excel to transform these ranked values into the standard normal cumulative distribution (mean of zero and a standard deviation of one). We calculated narrow sense heritabilities and additive genetic correlations using REML in the program ASREML. We report estimates based on sire variances and covariances because of the possibility of maternal and dominance effects that could compromise the analysis of dam components [51]. In the multivariate analyses, mating history (mated/unmated) was included as a fixed factor for all calculations of correlations involving female longevity.

Significance of heritabilities and genetic correlations were determined by testing the likelihood of the full model against the likelihood of the reduced or constrained model, with the difference of the likelihood values distributed as χ^2^ with 1 degree of freedom [51]. In the reduced form of the univariate analysis we removed the sire variance component from the model, leaving the dam component in the model as the only source of variance. In the bivariate analysis, the sire covariance was constrained to zero. As the covariance is the numerator in the equation for genetic correlations, by fixing it to zero we were able to test whether the correlation was significantly different from zero. When female lifespan was included in the analyses, female mating history (unmated, mated once, mated twice) was included in the model as a fixed factor if not stated otherwise.
